# Distributed gene clinical decision support system based on cloud computing

**DOI:** 10.1186/s12920-018-0415-1

**Published:** 2018-11-20

**Authors:** Bo Xu, Changlong Li, Hang Zhuang, Jiali Wang, Qingfeng Wang, Chao Wang, Xuehai Zhou

**Affiliations:** School of Computer Science and Technology, University of Science and Technology of China, Hefei, 230027 China

**Keywords:** Clinical decision support system, Cloud computing, Spark, Alluxio, Genetic data analysis, Read mapping

## Abstract

**Background:**

The clinical decision support system can effectively break the limitations of doctors’ knowledge and reduce the possibility of misdiagnosis to enhance health care. The traditional genetic data storage and analysis methods based on stand-alone environment are hard to meet the computational requirements with the rapid genetic data growth for the limited scalability.

**Methods:**

In this paper, we propose a distributed gene clinical decision support system, which is named GCDSS. And a prototype is implemented based on cloud computing technology. At the same time, we present CloudBWA which is a novel distributed read mapping algorithm leveraging batch processing strategy to map reads on Apache Spark.

**Results:**

Experiments show that the distributed gene clinical decision support system GCDSS and the distributed read mapping algorithm CloudBWA have outstanding performance and excellent scalability. Compared with state-of-the-art distributed algorithms, CloudBWA achieves up to 2.63 times speedup over SparkBWA. Compared with stand-alone algorithms, CloudBWA with 16 cores achieves up to 11.59 times speedup over BWA-MEM with 1 core.

**Conclusions:**

GCDSS is a distributed gene clinical decision support system based on cloud computing techniques. In particular, we incorporated a distributed genetic data analysis pipeline framework in the proposed GCDSS system. To boost the data processing of GCDSS, we propose CloudBWA, which is a novel distributed read mapping algorithm to leverage batch processing technique in mapping stage using Apache Spark platform.

## Background

Clinical decision support system (CDSS) provides clinicians, staff, patients, and other individuals with knowledge and person-specific information to enhance health and health care [1]. CDSS can effectively break the limitations of doctors’ knowledge and reduce the possibility of misdiagnosis to guarantee the quality of medical care with a lower medical expenses. Genetic diagnosis have the advantages of early detection, early discovery, early prevention and early treatment [2].

With the development of next-generation sequencing (NGS) technology, the number of newly sequenced data increase exponentially in recent years [3]. How to store and analyze the large amount of genetic data has become a huge challenge. Therefore, faster genetic data storage and analysis technologies are urgently needed. The current best practice genomic variant calling pipeline [4] is that use the Burrows-Wheeler Alignment tool (BWA) [5] to map genetic sequencing data to a reference and use the Genome Analysis Toolkit (GATK) [6] to produce high-quality variant calls, which takes approximately 120 h to process a single, high-quality human genome using a single, beefy node [7]. It need more time to compute when the sequencing depth is deeper or the length of reads is longer. What’s more, time is equal to life in the medical field, especially in emergency. Therefore, it is significant to accelerate the processing of genetic data for CDSS [8]. However, the traditional genetic data storage and analysis technology based on stand-alone environment are hard to meet the computational requirements with the rapid data growth for the limited scalability [2].

In order to solve the problems mentioned above, we propose GCDSS, a distributed gene clinical decision support system based on cloud computing technology. There are two main challenges in implementing GCDSS and improving its performance.

The first one is to design and implement a distributed genetic data analysis pipeline framework. The genetic data analysis usually involves a large amount of data, varied data formats and complicate analysis process. It is difficult to design and implement such framework. The second challenge is the limited scalability of traditional read mapping algorithms. Read mapping is the first and time-consuming step in the whole genetic data analysis pipeline. The lengths of the read are generally range from several to thousands of bases. A sample can typically produce billions of reads. It is critical and difficult for subsequent analysis to map these reads to the reference genome quickly and accurately.

In order to meet the challenges, we considered distributed storage, distributed computing framework and distributed algorithms. Also, we exploit cloud computing technology to parallelize genetic data analysis pipeline. We claim the following contributions and highlights:

1. In this paper, we design a distributed genetic data analysis pipeline framework for GCDSS and implements its prototype based on cloud computing technology. The unified pipeline framework effectively integrates read mapping and calibration, the variant discovery and genotyping, disease identification and analysis into the framework.
2. A novel distributed read mapping algorithm CloudBWA is presented in this paper. It enable traditional BWA-MEM [9] algorithms run in a horizontally scalable distributed environment based on Apache Spark. CloudBWA supports different genomics data formats, which facilitates the distributed storage of the large amount of genetic data. Also, we design and implement batch processing strategy to improve the performance of read mapping algorithm.

Our experimental result shows that GCDSS has an excellent scalability and an outstanding performance.

We first summarize the related work in following aspects:

A. *Genetic data analysis pipeline*

Over the past few years, a large number of distributed genetic data analysis pipeline frameworks have emerged in research institutions, such as Illumina [10], UCLA [11, 12], AMPLab [7, 13–15] and Broad Institute of MIT and Harvard [4, 12]. CS-BWAMEM [11], a fast and scalable read aligner at the cloud scale for genome sequencing, is developed by UCLA, which is for distributed read mapping, and implemented distributed sort and mark duplicates. Adam [7, 14], the distributed genomics data formats, and Avocado [13], the distributed variant discovery and genotyping algorithms, are presented by AMPLab. Moreover, Avocado has not implemented distributed read mapping. Distributed local sequence alignment algorithms DSW [16] and CloudSW [16] both achieve outstanding performance.

B. *Read mapping*

At present, BWA [5, 9, 17] is one of the best popular read mapping tool, which consists of BWA-SW [17], BWA-MEM [9] and BWA-backtrack [5]. SNAP, BWA and other traditional read mapping algorithms have a shortcoming of limited scalability. CS-BWAMEM is a fast and scalable read aligner at the cloud scale for genome sequencing, but it only support paired-end read mapping. SparkBWA is a tool that integrates the BWA [5, 9, 17] on a Apache Spark framework running on the top of Hadoop. Nevertheless, the I/O overhead of SparkBWA is extremely large because it has to read and wirte disk too many times [2]. SparkBWA can be error when numPartitions size is too large to run on Spark, such as the size of numPartitions is larger than the number of Spark workers. Moreover, if numPartitions size is relative small, it may result in uneven distribution of data and calculations, which ultimately reduce the performance of the system.

C. *Cloud computing*

Over the past decade or so, Hadoop [18], HDFS [19], Spark [20] and Alluxio [21] have been implemented and released as open source, which have greatly promoted the academic research and industrial applications. Especially in recent years, cloud computing technology has developed rapidly and become more mature, its performance has also been greatly improved [22–24]. Cloud computing has the characteristics of good fault tolerance, easy to expand, large scale, low cost and distributed, which has been widely used in academia and industry [25, 26]. However, the absorption of those technologies in the scientific field is slow [27].

## Methods

The GCDSS architecture includes the general overview, the workflow, the specific implementation and corresponding API respectively. This section especially put concentration on the design method and implementation for building the data analysis framework in distribution pipeline.

### System architecture

GCDSS uses genetic data for clinicians, patients, employees, and others to provide intelligent knowledge and personalized information to assist in clinical decision-making and help enhance health. The difference between GCDSS and traditional CDSS is that GCDSS mainly uses the genetic data to analyze and process, instead of using the data of traditional Chinese medicine or modern medicine to build the system. With the development of NGS technology, the number of newly sequenced data has exhibited an exponential increase in recent years. The traditional genetic data storage and analysis technology based on stand-alone environment have limited scalability, which has been difficult to meet the computational requirements of rapid data growth. This paper aims to design and implement a distributed gene clinical decision support system by using cloud computing technology.

**Fig. 1:**
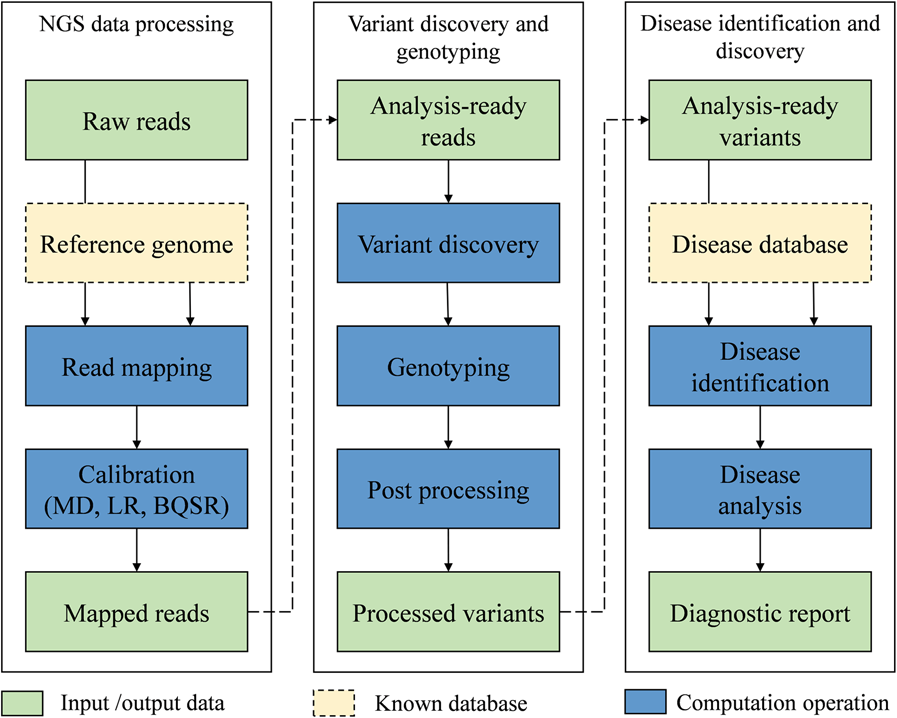
GCDSS workflow

As shown in Fig. 1, the GCDSS system workflow is composed of three stages: NGS data processing, variant discovery and genotyping, disease identification, and discovery. In this section, we will describe these stages respectively.

1. *The NGS processing*

The NGS data processing mainly consists of read mapping and calibration.

After achieving sequencing data from high-throughput sequencer like HiSeq X Ten of Illumina, GCDSS system needs to map billions of the raw reads to the reference genome. In the next step, it obtains the most probable location of every read, which is normally referred to read mapping. The Read mapping stage is generally complicated and critical for subsequent analysis, as it needs to accurately and efficiently map billions of reads to the reference genome. To address the scalability problem of traditional read mapping algorithms, we employ a distributed read mapping algorithm, named CloudBWA based on the cloud computing techniques. The CloudBWA will be described in detail in the next Section.

During the process of genetic sequencing by sequencer, errors during sample preparation and sequencing can lead to the duplication of some reads. To improve the accuracy of subsequent analysis, we detect duplicates by reads’ alignment position and orientation after read mapping, and the reads that have identical position and orientation in RDD [28] are assumed to be duplicates. All duplicate reads but the highest quality read are marked as duplicates. We can remove duplicate reads easily by filter function of Spark.

Since a lot of read mapping algorithms have adopted greedy strategy, which leads to inaccurate local alignment, it is necessary to LR for the inaccurate alignment after read mapping. The LR algorithm first identify regions as targets from reads, and then compute the convex hull of overlapping targets, and next classify reads and realignment reads of RDD.

During the process of genetic sequencing, systemic errors produced by the sequencer can lead to the incorrect assignment of base quality scores. BQSR is quite necessary for improving subsequent analysis accuracy. The correction is applied by estimating the error probability for each set of covariates under a beta-binomial model [7].

2) *Genotyping and variant discovery*

The second phase of the GCDSS workflow includes variant discovery and genotyping, both of which are intended to discover the possible variant in analysis-ready reads, as well as the genotyping procedure. Due to that the majority of variants is named single nucleotide polymorphism (SNP), and SNPs normally have stable heredity, wide distribution, and easy detection, therefore in this paper we mainly focus on the variant discovery and genotyping of SNP, and employ the insertion-deletion polymorphism (Indel) accordingly.

In this paper, we use Genotyping, which employs the biallelic genotyper [10] to obtain the sample genotype. Especially, it first marks the variants in the relevant variant RDD and gets the observations, and then for the next step, it turns the single variant observation into the genotype call, and finally creates a genotype RDD.

3) *Disease identification*

**Fig. 2:**
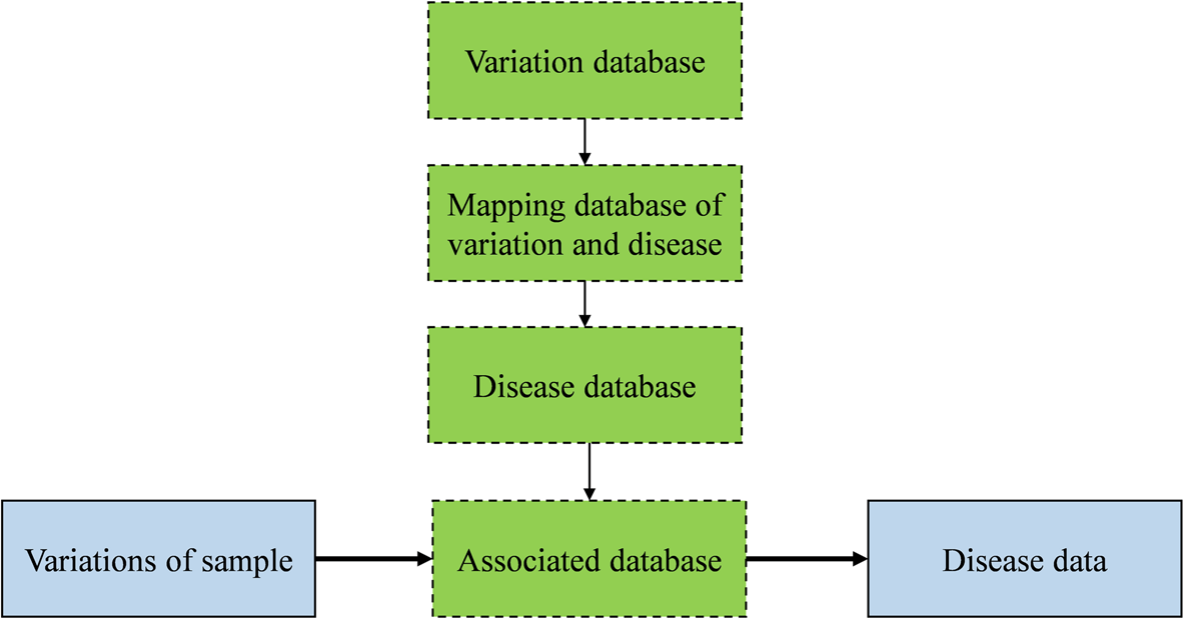
Workflow of disease identification and discovery

Finally, the last stage is the disease identification and discovery. As illustrated in Fig. 2, this disease identification phase is composed of two steps: the associated database construction and data analysis for the association.

The associated database construction stage mainly uses public database to build an associated database especially for the discovered variations with known diseases. It is acknowledged that NCBI has a public variation database whose size is as large as about 26 gigabytes with around 150 million variations [29]. In the databased, each variation contains the information with following items: the chromosome, position, variant id, reference base, alternate base. NCBI also provides a mapping database of disease and variation. The number of information in the mapping database is about 19,000. Each item contains the OMIM id, the locus id, the SNP, locus symbol, SNP id and so on. Meanwhile, the Online mendelian inheritance in man (OMIM) also provides a comprehensive and timely research support of human genes descriptions and phenotypes, as well as the relationships between them [30]. In this paper, we employ OMIM as disease database at present, due to that OMIM describes a wide variety of disease-related medical features, diagnosis measures, treatment measures, state-of-the-art research progress and other materials.

Also, the main steps to build an association database are listed as below:

1. Preprocessing and mapping the variation and disease database.
2. Analyzing the variation database comprehensively, then mapping the variation and disease database, filtering out the variations which are not related to disease.
3. Obtaining the association database by integrating the processed disease database, which should include the variation and corresponding disease information.

We provide two modes for association database. The simple mode only focuses on the disease id, position, chromosome name, reference base, variant id, and the alternate base. In addition to information of simple mode, the complex mode also includes the locus symbol, method, title, and the link to the corresponding description on the OMIM website. The link can be accessed to the latest OMIM website page with the latest information.

### System implementation

In this paper, GCDSS employs a hierarchical structure with cloud computing techniques. The system architecture of GCDSS consists of four layers: storage layer, computing layer, service layer and application layer.

#### Distributed storage

The first layer is the storage layer, which mainly in charges of storing the related data, including original genetic data, variant data, and disease data.

HDFS is designed to store and manage very large data sets. HDFS has the characteristics of high reliability, excellent scalability and high performance, which has been widely used in various fields. In GCDSS, HDFS is used as persistence in storage layer, which is mainly used to store large files or important data, including original sequencing data in FASTQ, result data in Adam format and so on.

However, the data in HDFS is stored in disk, which has limited I/O performance. To improve the read and write performance, we employ Alluxio as primary storage in the process of computing, which speeds up I/O performance by serving data from memory in local node rather than disks, and reduces network traffic between nodes by caching hot files in memory. Between different operations or Spark applications, GCDSS store intermediate data into Alluxio, which avoids storing intermediate data into disk and saves time.

In order to facilitate the calculation of Spark, we need to create several new RDDs by reading data from Alluxio or HDFS. To reduce repeat calculation in Spark application, we cache the RDD in memory by invoking persist or cache functions of Spark in different scenario. For example, we cache the data in memory while there are a series of transformations before an action.

After analyzing genetic data, we obtain result data and need show it to users. We employ NoSQL database Neo4j to show the result. Neo4j is a robust (fully ACID) transactional property graph database. It can vividly display the attributes of different things and their relationship and has high distributed query performance. We leverage Neo4j to store the result data, including information of the sample and its disease data, and provide query function for users.

To address the above problems, we leverage Adam system to convert traditional genetic data formats to Parquet format [31]. To be specific, the Columnar parquet formats can provide storage to minimize the I/O bandwidth and space [7].

#### Distributed computing framework

The computing layer is mainly responsible for analyzing genetic data and obtaining related results. Computing layer is based on Apache Spark, a memory-based distributed computing framework. Spark has the characteristics of excellent scalability and high performance. Spark is far more efficient than MapReduce [32] in memory, or 10x faster on disk. Spark provides different APIs in Java, Scala, and Python, which makes it easier to be used and compatible with other software like SAMtools [33].

BWA [5] is one of the most widely used read mapping algorithms. It has great accuracy and high performance in single node. BWA-MEM [9] is the newest algorithm in BWA tool. Therefore, we select BWA-MEM to read mapping. In this paper, we present CloudBWA, a distributed read mapping algorithm based on cloud computing technology. CloudBWA integrates BWA into Spark, which is based on Adam system for the genetic data formats, such as FASTA, FASTQ, SAM, and VCF, etc. [11]. Meanwhile We employ Avocado [10] Avocado to accomplish the variant discovery and genotyping.

#### Distributed algorithms

In order to facilitate the genetic data analysis in distribution, we implement several distributed algorithms, including the extract-transform-load library (ETLlib), the base algorithm library (BAlib), the conversion library (Clib) and the upload/download library (UPlib).

BAlib is a general algorithm library to distributed process and analyze the genetic data processing and analysis, including read mapping algorithm, Indel realignment algorithm, base quality score recalibration algorithm, mark duplicates algorithm, sorting algorithm, variants discovery algorithm, genotyping algorithm, disease identification and analysis algorithm and so on.

ETLlib is a library which is responsible for the extraction, cleaning, conversion and loading of genetic data, which is used to process the raw data and facilitates subsequent operations.

UPlib is a library dedicated to the uploading and downloading genetic data. As the usual FASTA, FASTQ and SAM data formats are not suitable for distributed environments at present, uploading files from the local file system requires related operations to process them. Moreover, it also requires related operations to process for downloading data from DFS to local file system.

Clib is a library designed for converting different data formats. It provides mutual conversion functions between standalone data formats FASTA, FASTQ, SAM, VCF and distributed data formats Adam [2].

## Distributed read mapping algorithm

In order to solve the scalability problems of the conventional read mapping algorithms, in this paper we propose a distributed read mapping algorithm CloudBWA, which is based on cloud computing techniques. This section describes the CloudBWA framework and the CloudBWA workflow respectively.

**Fig. 3:**
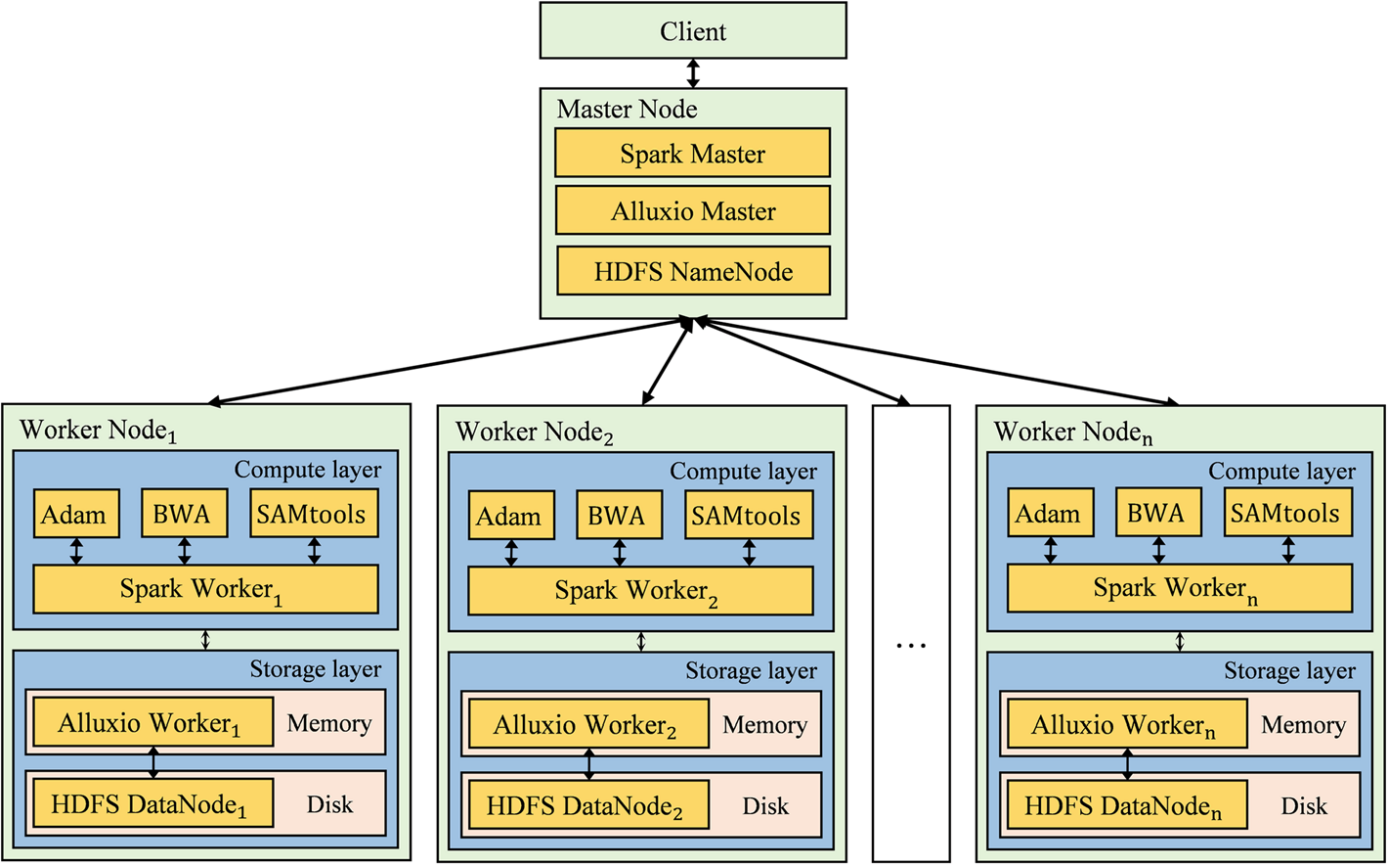
Framework of the CloudBWA algorithm

First, the CloudBWA framework is shown in Fig. 3, CloudBWA usually employs a Master-slave framework. In general, the Master node is primarily responsible for manage the metadata and the cluster, which combines Spark Master, Alluxio Master and HDFS NameNode. In comparison, the Slave node consists of two layers: a storage layer and a compute layer. The specifications of both layers are presented in Distributed read mapping algorithm section.

**Fig. 4:**
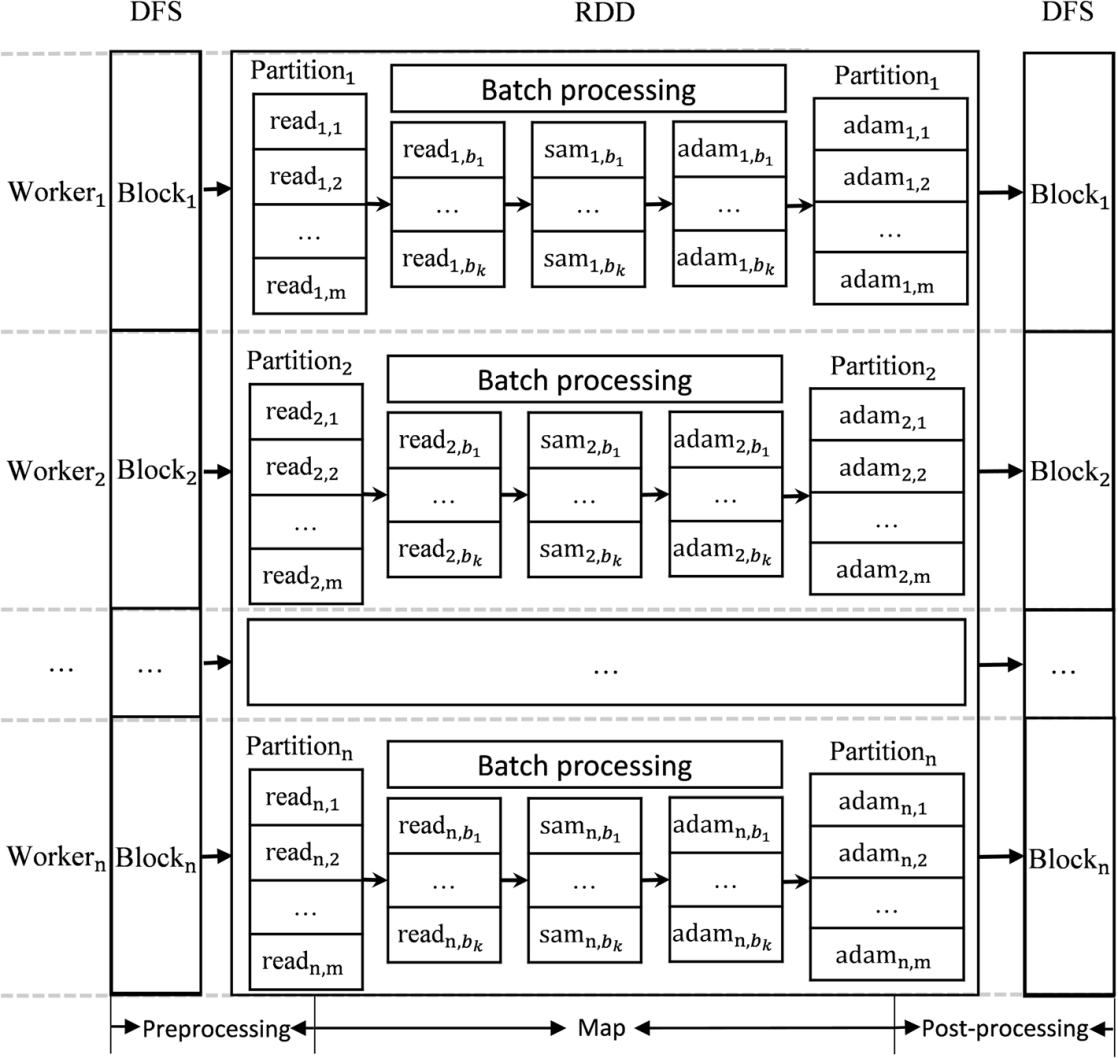
Workflow of the CloudBWA algorithm

The CloudBWA workflow mainly utilizes Spark and the BWA tools to a distributed read mapping framework, including data storage and conversion. As presented in Fig. 4, the CloudBWA algorithm is composed of three stages: the data preprocessing stage, the Map stage, and the post-processing stage.

1. *Preprocessing*

The preprocessing stage mainly reads data from the distributed file system (DFS) and works on the preprocess procedure. The DFS supported by CloudBWA has Alluxio and HDFS. Alluxio is small capacity and fast speed, and HDFS is more stable and larger capacity, but the read and write speed is relatively slower.

Data preprocessing contains following steps:

1. Inputting data. CloudBWA supports input genetic data in forms of the traditional FASTA, FASTQ and VCF formats, and then uses Adam to convert them into distributed data formats. CloudBWA also supports input genetic data in forms of Parquet data format.
2. Converting data. Since the Map phase requires information such as the sequence and name of the read, it is necessary to extract and convert.
3. Filtering data. Filtering out the data which is not in accordance with the requirements.
4. Pairing reads. It is necessary to pairing paired-end reads in preprocessing phase. Otherwise, it will increase the overhead of subsequent computation if paired-end reads are distributed in different nodes. CloudBWA pairs reads by invoking the Spark’s groupBy function and using the name of reads as key. Users can also specify the size of numPartitions, which can be used to adjust the number of partitions in each node and weaken the effect of stragglers.
5. Caching data. To reduce repeat computation, caching processed data into memory.
6. *Map*

This Map phase employs batch processing techniques to speed up the data processing procedure. When the batch is too small, the utilization rate of computing resources is not high. When the batch is too large, it will increase the system memory and CPU load and result in lower performance. So it is critical for the performance of CloudBWA to select the size of the batch.

As illustrated in Fig. 4, a new RDD of reads is generated after the preprocessing stage. Each RDD has several or a number of partitions. To facilitate the description comprehensively, each node in the Fig. 4 has only one partition, which can actually be adjusted by numPartitions in preprocessing phase. Considering that the data distribution is sufficiently uniform, each partition has m pairs of reads. Paired-end reads of Partition_1_are named as read_1, 1_ to read_1, m_. The paired-end reads in different partitions are generally different. CloudBWA provides two output mode: SAM and Adam mode. Assuming the size of batch is k. The Map phase are composed of following steps:

1. Loading the reference genome. The mapPartitions function of Spark is used to process each partition. The jBWA [34] is called to launch the reference genome that has already been built BWA index.
2. Read mapping stage. When the size of batch reaches k, the CloudBWA architecture will call the jBWA to do the read mapping process. If the remaining batch size of the partition is less than k, they will be mapped. For example, ${\mathrm{read}}_{1,{b}_1}$ will be processed and return to ${\mathrm{sam}}_{1,{b}_1}$ by read mapping. The ${\mathrm{sam}}_{1,{b}_1}$ is SAM format string.
3. Unifying the expression stage. Due to that jBWA may return multiple results after mapping, they need to be filtered and transformed. CloudBWA designs and implement a processing mechanism to unify the expression.
4. Processing in Adam mode. If the output is formatted as Adam mode, CloudBWA will invoke SAM tools to convert SAM format string into SAM Record format, and obtains or generates the reference sequence dictionary SQ, read group RG and program information PG, and then convert them into Adam format.
5. After processing an entire batch, the next batch of reads in the partition will be processed from (2) until the genetic data of the partition is processed.
6. When the whole partition is finished, CloudBWA will release the related data and operations of jBWA. When all the partitions are processed, CloudBWA will start the next phase automatically.
7. *Post processing*

CloudBWA needs to do a post processing after the map phase. The main steps are shown as follows:

1. Generating RDD. CloudBWA needs to obtain the reference sequence dictionary SQ, read group RG and program information PG, and then combines them with mapped reads in map phase to generate a new SAM or Adam RDD.
2. If the output mode is SAM mode, CloudBWA will save SAM RDD into DFS or return SAM RDD.
3. If the output mode is Adam mode, CloudBWA will return Adam RDD or save Adam RDD into DFS with specified storage block size and compression method.

Using Adam format output and storage increases computational overhead, but reduces storage space because Adam uses Parquet column storage, which can compress easily and has smaller storage space [2]. When the three phases are completed, CloudBWA completes the distributed read mapping.

## Results

The main goal of this paper is to solve the scalability problems of traditional genetic data analysis pipeline. In this section, we evaluate GCDSS in two major aspects: performance and scalability. CloudBWA is a distributed read mapping algorithm in GCDSS. The major aim of CloudBWA is address the second challenge: the scalability of traditional read mapping algorithms is limited. Read mapping is rather time-consuming in the whole genetic data analysis pipeline. Therefore, we focused on the evaluation of CloudBWA. We also evaluate the feasibility of the GCDSS prototype.

All our experiments are performed on an 8-node local cluster. The operation system of each node is Ubuntu-14.04.1. Each node has a dual core Intel Xeon W3505 CPU with 22GB of RAM, and it is connected via Gigabit Ethernet. The version of Apache Spark is 1.5.2. The Alluxio version is 1.3.0. The version of HDFS is 2.6.0. The version of Java JDK is 1.8.0_121. The version of Scala is 2.10.5. We employ wgsim [35] to generate simulation data. The version of wgsim is 0.3.2. The parameters of wgsim are set as default besides description. In order to validate algorithm or system in real environment, we use real datasets ERR000589 and SRR062634 (see Table 1) that are the same as SparkBWA [20]. ERR000589 has 23,928,016 reads and the length of each read is 51 base. SRR062634 has 48,297,986 reads and the length of each read is 100 base. Their sequencing platform is Illumina.

**Table 1:** Real datasets

Public datasets	Description	Number
GRCh38	Reference genome	About 3.2 billion base
ERR000589	Paired-end reads	23,928,016 reads
SRR062634	Paired-end reads	48,297,986 reads

CloudBWA is measured with different metrics, including the impact of different parameters on CloudBWA, the scalability evaluation, the performance comparison.

### Performance evaluation

We evaluated the impact of different parameters on CloudBWA, including the batch size and number of the Partitions.

1. Impact of the batch sizes and output mode

The experimental raw data reads are generated by wgsim with 20 million of reads at 50 base length. The memory of the Spark node is configured as 20G. The size of numPartitions is set to 32. Adam uses GZIP software to compress the data. Reference is chromosome 1 of GRCh38. We use HDFS as the distributed file system with FASTQ as the input format of reads.

**Fig. 5:**
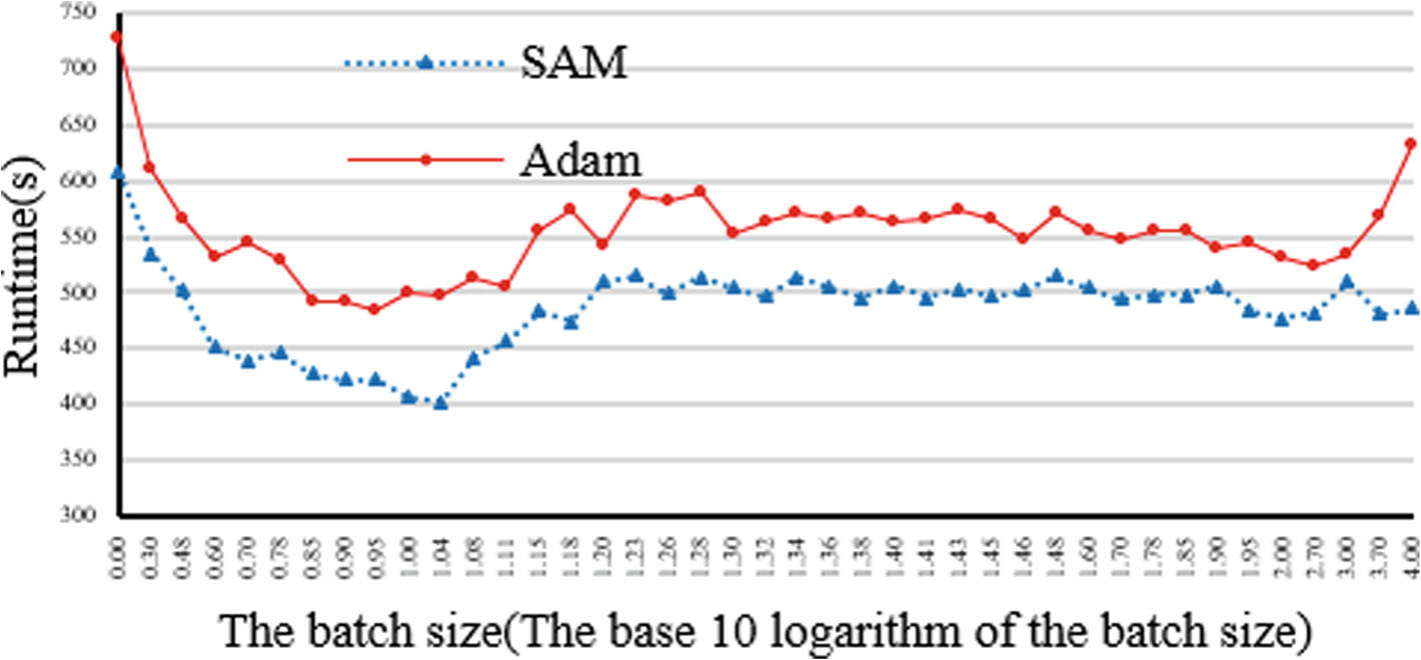
Impact evaluation of different batch size and output mode

Figure 5 illustrates impact evaluation of batch size and output mode on CloudBWA framework. The experimental result proposes that SAM mode is more efficient than the Adam mode for all possible batch sizes. This is due to that Adam format needs more computation resources, including the conversion and compression process. When batch size is only 1 read, the runtime of SAM and Adam mode is remarkable. When the batch size increases, the runtime of SAM mode will decrease at first and then increase accordingly, and finally become stable. The run time of Adam mode will rise when batch size is considerable. The runtime is the least when batch size is about 10 reads.

### Impact of different data format and numPartitions

CloudBWA supports both FASTQ and Adam input format. We use the same experimental data as the first experiment. The output mode is SAM mode with 10 reads for the batch size. We use Alluxio as the distributed file system with FASTQ as the input format of reads. The CloudBWA is evaluated with different numPartitions sizes.

**Fig. 6:**
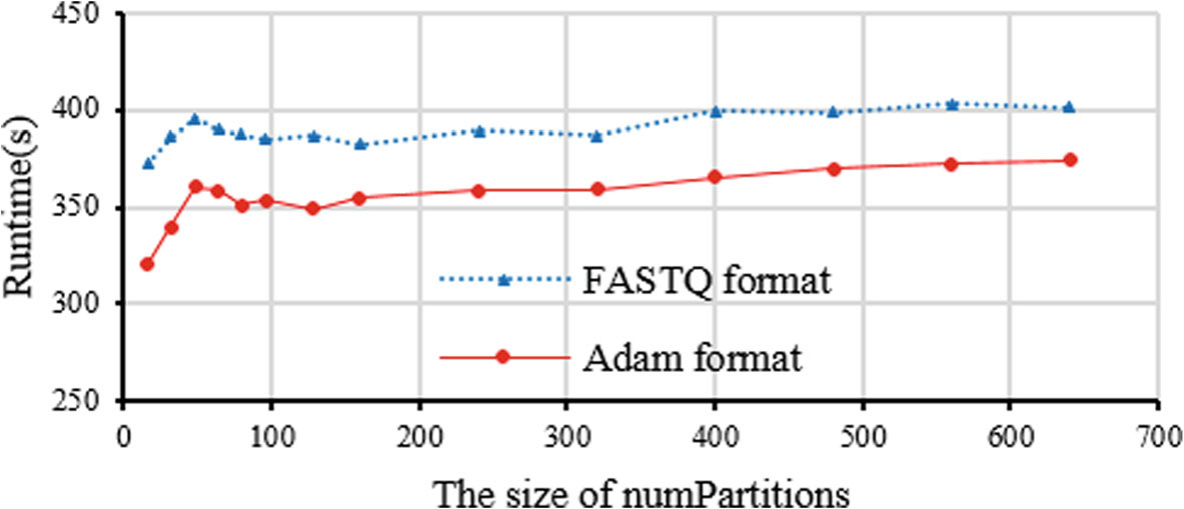
Impact evaluation of different input data format

Figure 6 demonstrates the impact of different input data formats on the CloudBWA. The experimental result illustrates that Adam input format is more efficient than FASTQ format. Adam format achieves 9.6% performance improvement over SAM format on average. When inputting data with Adam format in DFS, CloudBWA can directly process the data, which avoids extra transformation overhead like FASTQ format. The experimental result shows that with the growth in numPartitions, the running time of CloudBWA will grow quickly, and then becomes flat with minor increments. When the size of numPartitions is 16, the runtime of CloudBWA is the least. At present, CloudBWA employs BWA to read mapping in each partition, including loading index file and releasing related data.

### Scalability evaluation

In order to measure the scalability of CloudBWA, we run the algorithm with different number of nodes. The data are the same as the first experiment. Every node uses one core. The numPartitions size is 16 with Adam input data format. The output mode is SAM with 10 reads for the batch size.

**Fig. 7:**
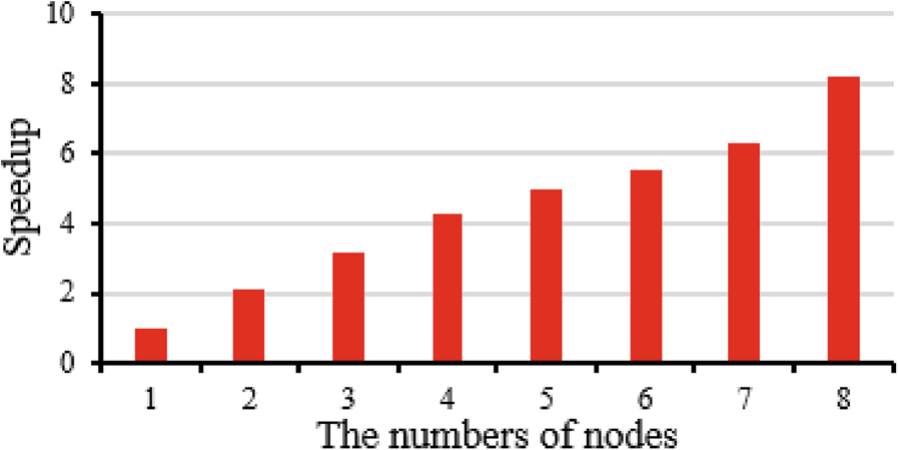
The speedup improvement by increasing the number of nodes

Figure 7 illustrates the speedup improvement of CloudBWA architecture in line with the number of nodes growth. The experimental result shows that CloudBWA achieves approximately linear speedup when the number of nodes grows from 1 to 8.

### Comparison

We use different experiments to compare the performance of CloudBWA and other related algorithms, including stand-alone algorithms and distributed algorithms.

#### Comparison with distributed algorithms

We compare CloudBWA with different distributed algorithms, including SparkBWA [20] and CS-BWAMEM [13], both of which are the state-of-the-art read mapping algorithms in distribution. The version of CS-BWAMEM is 0.2.2, with output format is Adam and 100 reads batch size. The version of SparkBWA is 0.2 with SAM output format and numPartitions size is 8 The SparkBWA uses two threads in each node. The version of CloudBWA is 1.0.1, with numPartitions size at 16 and batch size at 10 reads. The three algorithms use 8 nodes and 16 cores in cluster.

**Fig. 8:**
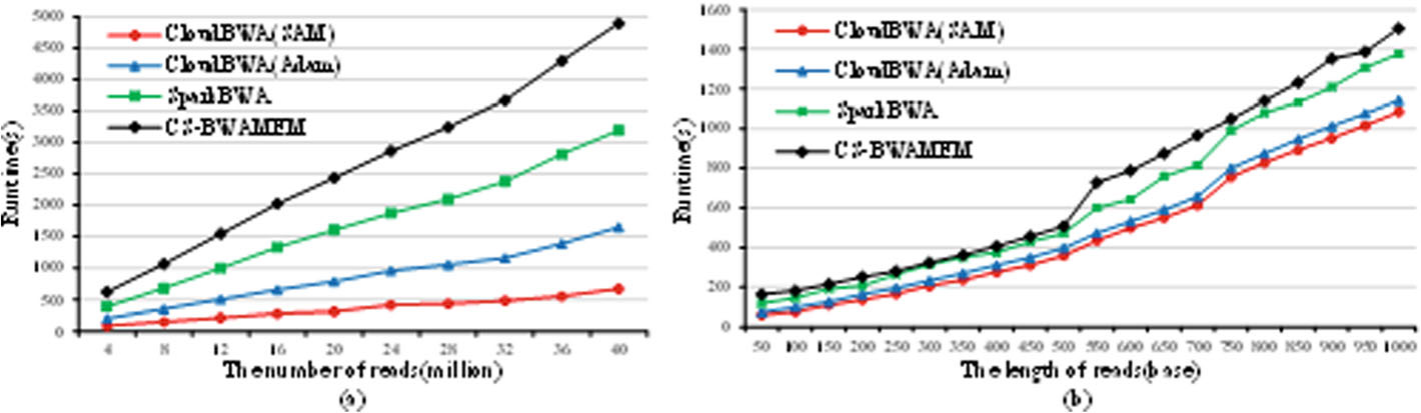
Performance comparison with distributed algorithms

Figure 8 illustrates the performance comparison with both distributed algorithms. In particular, Fig. 8a uses different number of reads, which is from 4 million to 40 million, and their length is 50 base. The experimental results demonstrate that CloudBWA is more efficient than the SparkBWA and CS-BWAMEM algorithm in different number of reads. The Adam mode of CloudBWA achieves average 1.84 times speedup over CS-BWAMEM. The SAM mode of CloudBWA gains up to 2.63 times speedup over SparkBWA. Figure 8b evaluates the results for different length of reads, which is from 50 to 1000 base. The numbers illustrate that CloudBWA is more efficient than SparkBWA and CS-BWAMEM in various length of reads. The Adam mode of CloudBWA gains up to 2.22 times speedup over CS-BWAMEM. The SAM mode of CloudBWA achieves 1.44 times speedup over SparkBWA on average.

#### Comparison with real data

In order to more fully validate CloudBWA, we design two different experiments with real data (see Table 1).

The stand-alone algorithms use one node and one core. The distributed algorithms use 8 nodes with 16 cores. The version of CloudBWA is 1.2.0. CloudBWA uses 160 as NumPartitions size and 11 as batch size for ERR000589. CloudBWA uses 128 as NumPartitions size and 10 reads as batch size for SRR062634. We cannot obtain experiment result of CS-BWAMEM because of it has out of memory error in real data.

**Fig. 9:**
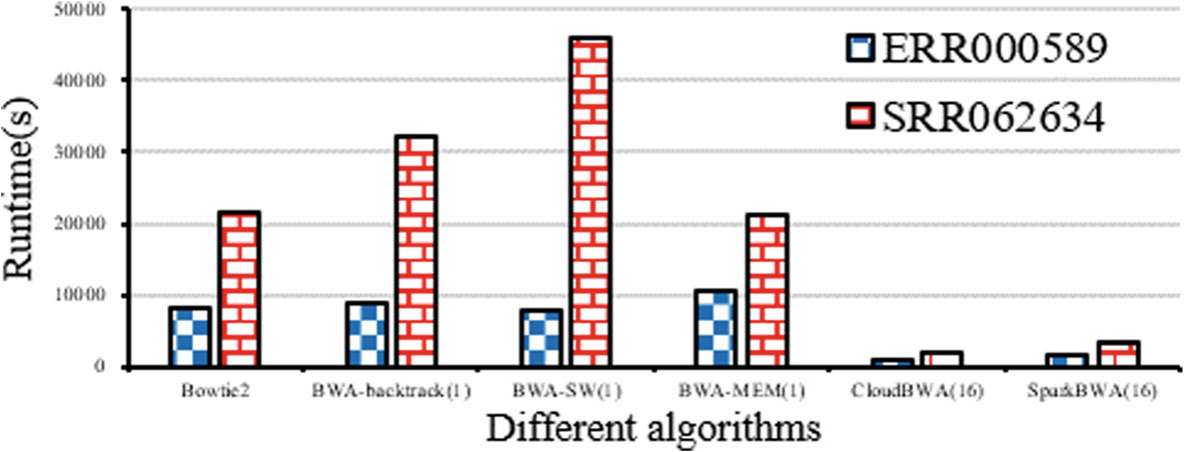
Performance comparison with real data

Figure 9 illustrates the performance comparison with real data. The parameter of parentheses is the number of processor cores. The experimental result illustrates that CloudBWA has outstanding performance. For ERR000589, CloudBWA (16) achieves 11.59, 8.39, 1.66 times speedup over BWA-MEM (1), BWA-SW (1), SparkBWA (16), respectively. For SRR0062634, CloudBWA (16) achieves 11.02, 23.86, 1.68 times speedup over BWA-MEM (1), BWA-SW (1), SparkBWA (16), respectively.

## Discussion

After evaluating the CloudBWA, which is the key component algorithm in GCDSS, we also measure the other GCDSS’s components. We especially focus on the performance and scalability analysis.

### Calibration evaluation

The experimental benchmarks and configurations are the same as the experiment of A 3) (1). The length of reads is set to 50.

#### Performance of calibration

**Fig. 10:**
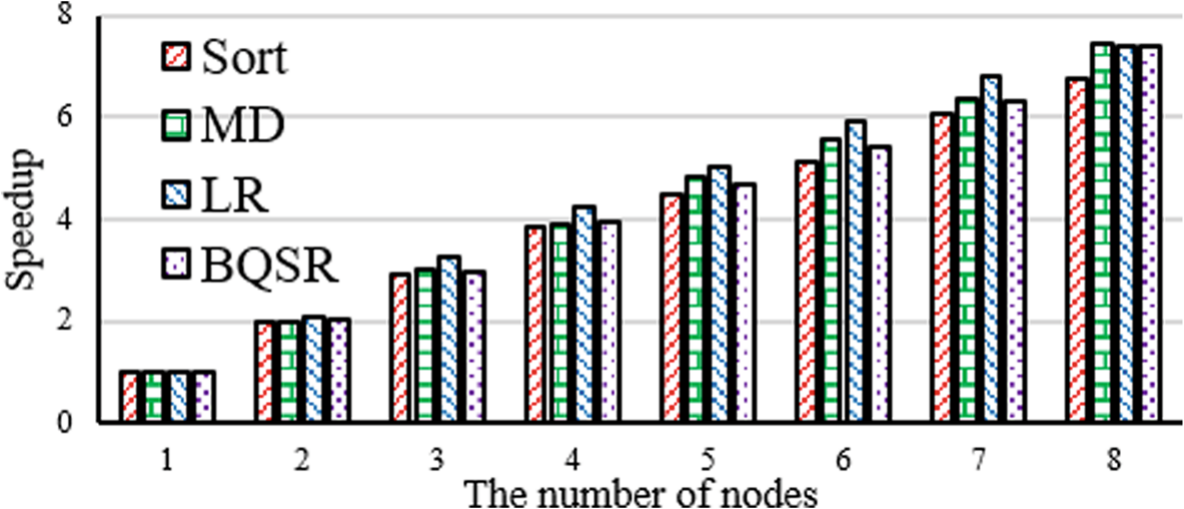
Performance evaluation of calibration

Figure 10 illustrates the performance of calibration. The experimental results demonstrate that sort is the fastest process and LR is the slowest process in the calibration procedure. MD is actually slower than BQSR at the beginning, but MD is far more efficient when the number of reads is more than 20 million. With the growth in the population of reads, the four operations’ runtime of calibration are also approximately linear increasing.

#### Scalability of calibration

**Fig. 11:**
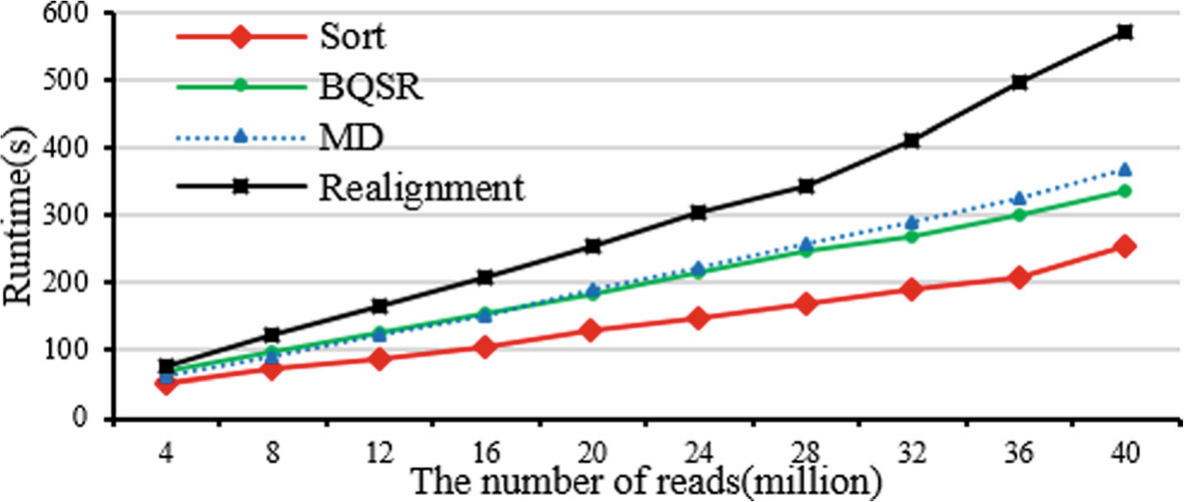
Scalability evaluation of calibration

Figure 11 demonstrates the scalability of calibration. The experimental result reveals that the four operations of calibration achieve about linear speedup when the number of nodes increases from 1 to 8 in cluster.

### Variant discovery and genotyping evaluation

The experimental benchmarks and configurations are the same as the experiment of A 3) (1). The length of reads is set to 50.

#### Performance evaluation

**Fig. 12:**
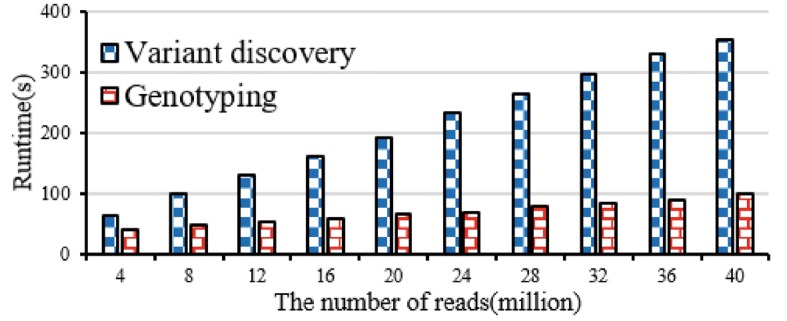
Performance evaluation of variant discovery and genotyping

Figure 12 illustrates performance of variant discovery and genotyping. The experimental result shows that with the growth of the number of reads, the runtime of variant discovery and genotyping both are around near linear.

#### Scalability evaluation

**Fig. 13:**
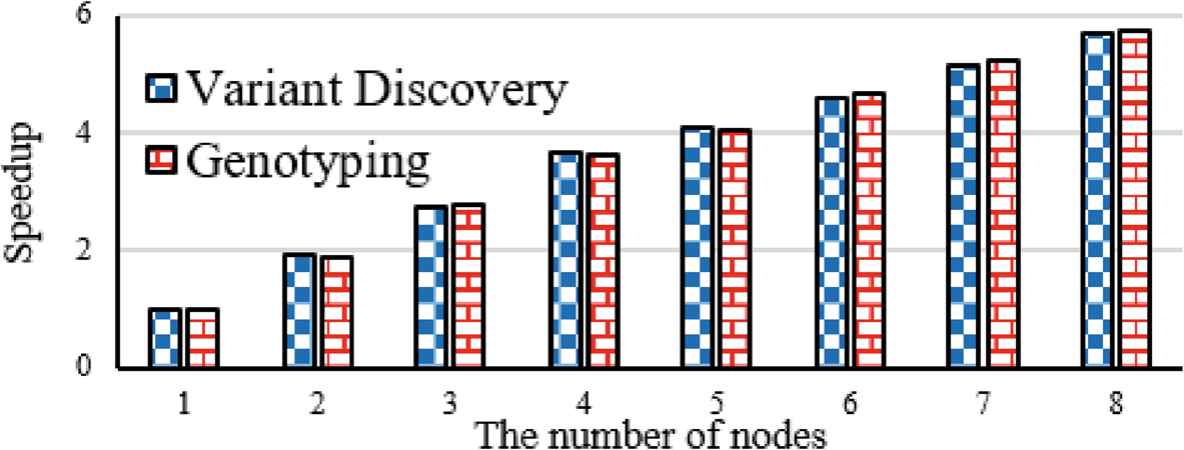
Scalability evaluation of variant discovery and genotyping

Figure 13 tells scalability of variant discovery and genotyping. The experimental result shows that variant discovery and genotyping both achieve near linear speedup in line with the growth the number of nodes from 1 to 8.

### Disease identification and discovery evaluation

Disease identification and discovery also plays a vital role of the GCDSS. We also accomplish several experiments to measure the disease identification and discovery. In order to analyze the GCDSS system comprehensively, this paper also incorporates the benchmarks same as the previous read mapping and variant analysis.

Table 2 tells the evaluation results of disease identification and discovery. We select 4 million, 20 million, and 40 million respectively. The experiment result shows that the corresponding number of data are 3, 14, and 34 after the data are analyzed by disease identification and discovery of GCDSS.

**Table 2:** Evaluation of disease identification and discovery

Number of raw reads	4,000,000	20,000,000	40,000,000
Number of mapped reads	3,911,329	19,553,895	39,107,115
Number of mate mapped reads	3,824,558	19,117,950	38,234,922
Reads number after variant discovery	3,655,139	17,772,692	37,313,251
Reads number after genotyping	5071	13,797	15,571
Reads number after disease analysis	3	14	34

## Conclusions

In this paper, we have presented GCDSS, which is a distributed gene clinical decision support system based on cloud computing techniques. In particular, we incorporated a distributed genetic data analysis pipeline framework in the proposed GCDSS system. To boost the data processing of GCDSS, we propose CloudBWA, which is a novel distributed read mapping algorithm to leverage batch processing technique in mapping stage using Apache Spark platform. The experimental results show that CloudBWA is able to achieve outstanding performance with excellent scalability. Compared to state-of-the-art distributed read mapping algorithms, CloudBWA achieves up to 2.63 times speedup over SparkBWA. Compared with unicore read mapping algorithms, CloudBWA with 16 cores achieves up to 11.59 times speedup over BWA-MEM with uniprocessor. We also evaluated other GCDSS’s key components, including calibration, variant discovery, genotyping, disease identification, and discovery. The experimental results illustrate that GCDSS also achieves remarkable speedup with satisfying scalability.

In the future, we plan to exploit numerous technologies to further improve performance and increase the variety of disease analysis.

### Availability

An open source GCDSS (under GNU GPL v.2 license) are available at: https://github.com/xubo245/GCDSS.
